# Diagnostic performance of dedicated breast positron emission tomography

**DOI:** 10.1007/s12282-022-01381-x

**Published:** 2022-06-29

**Authors:** Rikako Hashimoto, Sadako Akashi-Tanaka, Chie Watanabe, Hiroko Masuda, Kanae Taruno, Tomoko Takamaru, Yoshimi Ide, Takashi Kuwayama, Yasuhiro Kobayashi, Masafumi Takimoto, Seigo Nakamura

**Affiliations:** 1 Division of Breast Surgical Oncology, Department of Surgery, Showa University School of Medicine, 1-5-8 Hatanodai, Shinagawa, Tokyo 142-8666 Japan; 2 Tokyo Midtown Clinic, Midtown Tower 6F, Akasaka 9-7-1, Minato, Tokyo 107-6206 Japan; 3 Department of Pathology, Showa University School of Medicine, 1-5-8 Hatanodai, Shinagawa, Tokyo 142-8666 Japan

**Keywords:** Breast cancer, F-18 fluorodeoxyglucose, Dedicated breast positron emission tomography, Whole-body positron emission tomography, Magnetic resonance imaging, Standardised uptake value

## Abstract

**Background:**

Dedicated breast positron emission tomography (dbPET) has been developed for detecting smaller breast cancer. We investigated the diagnostic performance of dbPET in patients with known breast cancer.

**Methods:**

Eighty-two preoperative patients with breast cancer were included in the study (84 tumours: 11 ductal carcinomas in situ [DCIS], 73 invasive cancers). They underwent mammography (MMG), ultrasonography (US), and contrast-enhanced breast magnetic resonance imaging (MRI) before whole-body PET/MRI (WBPET/MRI) and dbPET. We evaluated the sensitivity of all modalities, and the association between the maximum standard uptake value (SUVmax) level and histopathological features.

**Results:**

The sensitivities of MMG, US, MRI, WBPET/MRI and dbPET for all tumours were 81.2% (65/80), 98.8% (83/84), 98.6% (73/74), 86.9% (73/84), and 89.2% (75/84), respectively. For 11 DCIS and 22 small invasive cancers (≤ 2 cm), the sensitivity of dbPET (84.9%) tended to be higher than that of WBPET/MRI (69.7%) (*p* = 0.095). Seven tumours were detected by dbPET only, but not by WBPET/MRI. Five tumours were detected by only WBPET/MRI because of the blind area of dbPET detector, requiring a wider field of view. After making the mat of dbPET detector thinner, all 22 scanned tumours were depicted. The higher SUVmax of dbPET was significantly related to the negative oestrogen receptor status, higher nuclear grade, and higher Ki67 (*p* < 0.001).

**Conclusions:**

The sensitivity of dbPET for early breast cancer was higher than that of WBPET/MRI. High SUVmax was related to aggressive features of tumours. Moreover, dbPET can be used for the diagnosis and oncological evaluation of breast cancer.

## Introduction

Breast cancer is the most common cancer among women in Japan and worldwide. The morbidity associated with breast cancer in Japan is steadily increasing. The onset of breast cancer typically occurs at the age of 40–49 years in Asian countries, including Japan [1]. Younger women tend to have dense breast with less fat tissue compared with older women. Dense breast is one of the factors that reduce the sensitivity of mammography (MMG) in detecting breast cancer. There are some reports on extremely dense breast tending to have a higher risk of developing breast cancer than fatty breast [2]. Therefore, improving the accuracy of diagnostic imaging in dense breasts is a major challenge that must be urgently addressed.

Whole-body positron emission tomography (WBPET) using F-18 fluorodeoxyglucose (FDG) is widely used to evaluate the whole body for determining the stage and progression of breast cancer. Furthermore, the FDG uptake level is significantly associated with pathological and immunohistochemical factors [3–8]. However, WBPET is known to have a lower detection rate for smaller and lower grade breast cancers because of the limitation of spatial resolution and FDG uptake.

Breast positron emission tomography (PET) has been developed to detect early breast cancer and has been reported to be able to detect tumours sized < 1 cm, including both invasive cancers and ductal carcinomas in situ (DCIS), which are difficult to detect with WBPET [9–11]. Breast PET is classified into the following two types: the opposite type (positron emission mammography; PEM) and ring-shaped type (dedicated breast PET; dbPET) [12]. In PEM, the breast is compressed as in the scanning approach in an X-ray MMG. Using this type, it is possible to obtain multiple plane slices with a mobile detector in the sitting position. In contrast, dbPET with circumferentially arranged detectors enables imaging of the whole breast in the prone position, comparable to breast magnetic resonance imaging (MRI), and generates maximum-intensity projection images. Nishimatsu et al. [13] published a study comparing the sensitivity and specificity of dbPET and WBPET/computed tomography (CT). The lesion-based sensitivities of dbPET and WBPET/CT were 92% and 88%, respectively (*p* = 0.06). Moreover, dbPET was covered by the Japanese medical insurance in July 2013 on the condition that dbPET was performed on the same day as WBPET. In this study, we used the dbPET that was developed in Japan.

We investigated the imaging sensitivity of dbPET for breast cancer. For practical clinical application, we compared the sensitivity of dbPET with that of MMG, ultrasonography (US), contrast-enhanced MRI, and WBPET/MRI. We also analysed the association between the FDG uptake level on dbPET and histopathological features.

## Patients and methods

### Study design

This study was a combined effort of Showa University, Midtown Clinic, and Shimadzu Corporation. All patient recruitments were from Showa University Hospital between May 2015 and December 2016. Patient recruitment was continued during the study period. This study had a prospective design, and informed consent was obtained from all participants. However, the study was interrupted for approximately 6 months to improve the equipment mat described later. All WBPET/MRI and dbPET scanning were performed at Midtown Clinic. This study was conducted in accordance with the ethical principles of the Declaration of Helsinki and the ethical guidelines for human medical research (approved on 22 December 2014 by the Ministry of Health, Labour, and Welfare), was approved by the Institutional Review Board of Showa University Hospital (Approval number 1723), and registered on the University Hospital Medical Information Network (UMIN ID 000027227).

### Patients

Patients who had been diagnosed with breast cancer but had not commenced treatment were included in the study. In total, 82 Japanese women with histologically diagnosed breast cancer (84 tumours) consented to participate in this study. In particular, 27 patients (33%) received some form of cancer treatment after scanning dbPET and WBPET/MRI. All patients underwent US, WBPET/MRI, and dbPET. Seventy-eight patients (80 tumours) underwent MMG and 70 (74 tumours) underwent MRI. All modalities were performed prior to the treatment of all patients.

### Imaging protocol

All patients underwent WBPET/MRI using Biograph mMR^®^ (Siemens Healthcare, Erlangen, Germany), followed by dbPET using the Elmammo^®^ dbPET system (Shimadzu, Kyoto, Japan). WBPET/MRI was performed at 60 min after an F-18 FDG (3.0 MBq/kg) injection. First, WBPET/MRI was performed in the supine position, and breast PET/MRI with breast coil was performed in the prone position (magnetic resonance attenuation correction: volumetric interpolated breath-hold examination; 3-mm slice; repetition time, 3 ms; echo time, 1.03 ms; fractional anisotropy, 10; field of view [FOV], 450 mm). The total scanning time of WBPET/MRI and breast PET/MRI was approximately 60 min (scan time, 4 min/bed; image reconstruction: point-spread function; iteration, 3; subset, 21; filter, 4 mm; FOV, 420 mm; matrix, 172). The reason for the performance of additional breast PET/MRI in the prone position was to investigate the blind area of dbPET. The sensitivity of breast cancer by WBPET/MRI in this study was comprehensively evaluated using breast PET/MRI. The dbPET was performed 120 min after the F-18 FDG injection, and the emission time was 5 min per breast in the prone position. The detector consisted of four layers of a 32 × 32 cerium-doped lutetium gadolinium oxy-orthosilicate crystal array (crystal size, 1.44 × 1.44 × 18 mm), a light guide, and a 64-channel position-sensitive photomultiplier tube. The FOV was 185 × 156.5 mm. We initially used a 20-mm-thick mat on the detector. However, we changed the thickness of the mat from 20 to 5 mm, because there were some undetected tumours in the blind areas.

We compared the sensitivities of dbPET with those of the existing breast cancer modalities, including US, MMG, MRI, and WBPET/MRI. The US and MMG images were evaluated based on the guidelines for breast US [14] and mammography [15], respectively, by breast surgeons and radiologists at Showa University. Breast-enhanced MRI images were evaluated using the Breast Imaging Reporting and Data System (BI-RADS^®^) [16] by radiologists at Showa University. All PET images (WBPET/MRI and dbPET) were interpreted by one radiologist with over 6 years of experience as a nuclear medicine and diagnostic imaging specialist. The radiologist recognised in advance that the patients had breast cancer. However, the interpretations of WBPET/MRI and dbPET were performed without detailed information. At the study initiation, there was no consensus on the interpretation method for dbPET. Therefore, we regarded the lesion with SUVmax of ≥ 1 and the morphology suggesting a mass or segmental non-mass FDG uptake as the abnormal uptake. We considered cases with strong background breast fibroglandular uptake (bFGU) as those with an abnormal uptake, which was conspicuous from the bFGU.

### Pathological diagnosis

In this study, we used histopathological information, which was obtained using needle biopsy before surgery or any drug treatments. We evaluated the histopathological features using histology, nuclear grade (NG), oestrogen receptor (ER) status, progesterone receptor (PgR) status, human epidermal growth factor 2 (HER2) status, and Ki67 labelling index (Ki67%). ER, PgR, and HER2 statuses were determined using the American Society of Clinical Oncology/College of American Pathologists guidelines for breast cancer [17, 18].

### Statistical analysis

As this was an observational, prospective study, no formal sample size calculation was performed. The results are presented as the number and proportion of eligible populations. We analysed the characteristics of patients and clinicopathological features in the detected and undetected tumour groups using Pearson’s chi-squared test, Fisher’s exact test, and analysis of variance (ANOVA). The McNemar test was performed for the comparison of sensitivities. To analyse the relation between SUVmax and clinicopathological features, we used ANOVA and the *t* test. The SUVmax is expressed as mean ± standard deviation. We used the JMP professional software, version 15.0 (SAS Institute, Cary, NC, USA), for statistical processing. Statistical significance in all statistical tests was set at *p* < 0.05.

## Results

The characteristics of the 82 patients (84 tumours) in this study are summarised in Table 1.

**Table 1:** Characteristics of 84 breast cancers among 82 patients with breast cancer

	Dedicated breast PET			
	Total *n* = 84	Detectable *n* = 75 (%)	Undetectable *n* = 9 (%)	*p* value^a^
Median age (years) (range)	50 (32–85)	50 (32–85)	51 (35–70)	0.73
Mean body mass index (kg/m^2^)	22.3	23.12	20.35	0.099
Distant metastasis	4	4 (100)	0 (0)	
No distant metastasis	80	71 (88.7)	9 (11.3)	
*Clinical tumour status*				
T is	11	9 (81.8)	2 (18.2)	0.875
1	45	38 (84.4)	7 (15.6)	
2	20	20 (100)	0 (0)	
3–4	8	8 (100)	0 (0)	
*Histology*				
Ductal carcinoma in situ	11	9 (81.8)	2 (18.2)	0.0004
Invasive ductal carcinoma	69	64 (92.7)	5 (7.3)	
Invasive lobular carcinoma	2	0 (0)	2 (100)	
Special types (mucinous carcinoma, apocrine carcinoma)	2	1 (50)	1(50)	
*Nuclear grade*				
1	52	45 (86.5)	7 (13.5)	0.168
2	11	9 (81.8)	2 (18.2)	
3	21	21 (100)	0 (0)	
*Oestrogen receptor* [17]				
Positive (≥ 10%)	65	56 (86.2)	9 (13.8)	0.112
Negative (< 10%)	19	19 (100)	0 (0)	
*HER2* [18]				
Positive (score 3 or 2 with FISH)	17	16 (94.1)	1 (5.9)	1
Negative (score 1 or 2)	67	59 (88.1)	8 (11.9)	
*Ki67 (%)*				
< 20	31	27 (87.1)	4 (12.9)	0.516
20–50	29	26 (89.7)	3 (10.3)	
> 50	24	22 (91.7)	2 (8.3)	

dbPET, dedicated breast positron emission tomography; FISH, fluorescence in situ hybridisation; HER2, human epidermal growth factor receptor 2

^a^Pearson’s chi-squared test, Fisher’s exact test, and ANOVA

The median age of the patients included in this study was 50 (range, 32–85) years. The body mass index (BMI) tended to be higher in the detected than in the undetected group with no significant difference (*p* = 0.09). Four patients were diagnosed with stage IV breast cancer using WBPET/MRI. Eighty tumours (95.2%) were operable. Moreover, 27 patients (33%) received some form of cancer treatment after dbPET and WBPET/MRI. Seventy-five tumours (89.3%) were detected by dbPET but nine tumours (10.7%) were undetected. The dbPET detection rates of histological types in DCIS and invasive ductal carcinoma (IDC) cases were 81.8% and 92.7%, respectively. The two invasive lobular carcinoma (ILC) cases as well as the mucinous carcinoma case were undetected by dbPET. No significant differences were observed using dbPET between the detected and undetected groups in the clinical T stage (clinical size of the tumour), NG, ER, HER2 status, and Ki67% of the target tumours.

**Fig. 1:**
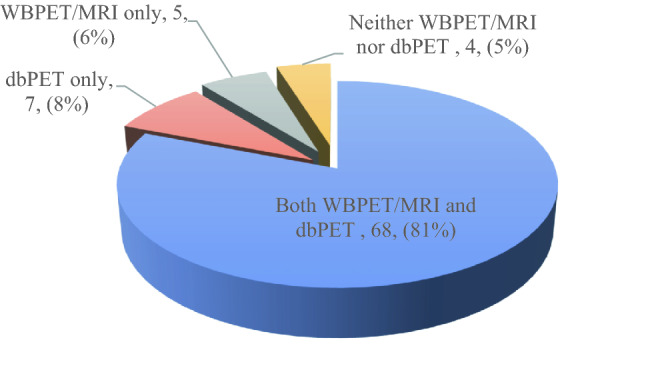
Detection rate of dbPET and WBPET/MRI (*n* = 84). Five tumours were detected by WBPET/MRI only and were suspected to be in the blind area of dbPET. dbPET, dedicated breast positron emission tomography; WBPET/MRI, whole-body positron emission tomography/magnetic resonance imaging

We compared the detected rates of dbPET and WBPET/MRI for all tumours, as shown in Fig. 1. Of 84 tumours, 68 (81%) were detected by both dbPET and WBPET/MRI. Neither dbPET nor WBPET/MRI detected four tumours (5%). Seven tumours (8%) were detected by dbPET only, but not by WBPET/MRI. The tumours included four cases of DCIS and three very small IDC cases. The details of those tumours detected only by dbPET are summarised in Table 2.

**Table 2:** Tumours that could only be visualised with dbPET, but not with WBPET/MRI

	dbPET (SUVmax)	Histological type	Maximal invasive diameter (ductal spread) (mm)	Ki67 (%)	NG	ER (%)	HER2 score
1	4.08	DCIS	0 (50)	10–20	1	> 90	1
2	3.07	DCIS	0 (17)	10–20	1	0	3
3	2.76	DCIS	0 (40)	40–50	1	> 90	1
4	2.1	DCIS	0 (9)	40–50	2	> 90	1
5	5.8	IDC	9	20–30	1	> 90	1
6	2.15	IDC	4	5–10	1	> 90	1
7	1.82	IDC	3	40–50	2	> 90	1

dbPET, dedicated breast positron emission tomography; HER2, human epidermal growth factor receptor 2; WBPET/MRI, whole-body positron emission tomography/magnetic resonance imaging; DCIS, ductal carcinoma in situ; IDC, invasive ductal carcinoma; NG, nuclear grade; ER, oestrogen receptor

**Fig. 2:**
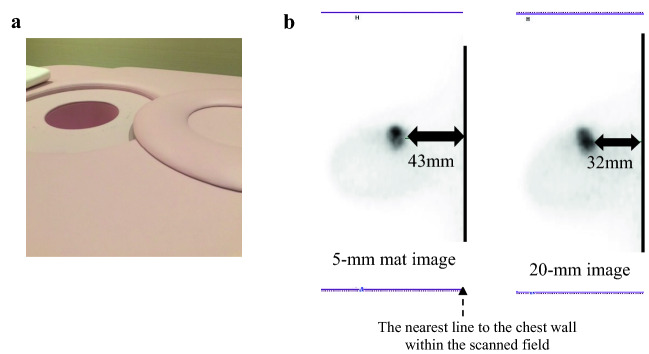
Reducing the blind area of dbPET.** a** Detachable 20-mm mat and dbPET detector. **b** Right image: a sagittal view of the dbPET scanner using a 20-mm mat. The distance from the limit end of the chest wall was 32 mm; left image: a sagittal view of the dbPET scanner using a 5-mm mat. The distance from the limit end of the chest wall was 43 mm. Reducing the mat thickness was effective in reducing the blind area close to the chest wall. dbPET, dedicated breast positron emission tomography

Five tumours (6%) were detected by WBPET/MRI only. Those tumours were located in the blind area of the dbPET detector. The out-of-field tumours triggered the need for improvement of the dbPET mat. Thus, we changed the mat thickness from 20 to 5 mm. After the reduction of the mat thickness, we restarted the study and scanned 22 patients. None of the 22 tumours were missed (Fig. 2).

Table 3 shows the sensitivity of each modality for 11 DCIS, 33 early breast cancers (11 DCIS and 22 small invasive cancers; invasive diameter, ≤ 20 mm), and all 84 tumours. US and MRI had the highest overall sensitivity (98.8% and 98.6%, respectively) for all breast cancers. For 11 DCIS, dbPET and MMG had the same sensitivity (81.8%), which was higher than that of WBPET/MRI (54.5%) without significant difference (*p* = 0.179). For 33 early breast cancers, the sensitivity of dbPET (84.9%) tended to be higher than that of WBPET/MRI (69.7%) and MMG (75.8%) but with no statistically significant difference (*p* = 0.095 and *p* = 0.365, respectively).

**Table 3:** Target lesion imaging sensitivity (%) by imaging modality

Modality	DCIS *n* = 11	Early breast cancers; DCIS + small invasive carcinomas (Invasive diameter ≤ 2 cm^a^) *n* = 33	All carcinomas *n* = 84
dbPET	81.8 (9/11)	84.9 (28/33)	89.2 (75/84)
WBPET/MRI	54.5 (6/11)	69.7 (23/33)	86.9 (73/84)
MRI	90.9 (10/11)	96.9 (32/33)	98.6 (73/74)
US	90.9 (10/11)	96.9 (32/33)	98.8 (83/84)
MMG	81.8 (9/11)	75.8 (25/33)	81.2 (65/80)

dbPET, dedicated breast positron emission tomography; HER2, human epidermal growth factor receptor 2; WBPET/MRI, whole-body positron emission tomography/magnetic resonance imaging; DCIS, ductal carcinoma in situ

^a^The size was confirmed by surgical specimens and the excluded tumours received neoadjuvant chemotherapy

We analysed the association between the SUVmax of dbPET and the clinicopathological characteristics (Table 4). The SUVmax level tended to increase as the tumour diameter increased. However, there was no significant difference between the SUVmax and maximum infiltration diameter (*p* = 0.211), as well as between the HER2 status and SUVmax (*p* = 0.149). In contrast, the higher SUVmax of dbPET was significantly related to the negative ER status (*p* = 0.0001), higher NG (*p* = 0.0001), and higher Ki67 (*p* = 0.0002).

**Table 4:** Association between clinicopathological characteristics and SUVmax

	(*n*)	SUVmax (mean ± SD)^a^	*p* value^a^
Tumour invasive size^b^ (mm) (*n* = 57)	< 10 (20)	4.9 ± 5.0	0.211
	10–20 (25)	8.5 ± 5.8	
	> 20 (12)	12.6 ± 11.0	
ER (%)	Negative (19)	20.9 ± 11.5	0.0001
	Positive (65)	9.0 ± 7.9	
HER2	Negative (68)	11.0 ± 9.8	0.149
	Positive (16)	15.2 ± 11.3	
NG	1 (52)	8.4 ± 7.7	0.0001
	2 (11)	10.2 ± 8.9	
	3 (21)	20.5 ± 11.1	
Ki67 (%)	< 20 (30)	6.5 ± 5.0	0.0002
	≥ 20 (54)	14.7 ± 11.1	

dbPET, dedicated breast positron emission tomography; HER2, human epidermal growth factor receptor 2; WBPET/MRI, whole-body positron emission tomography/magnetic resonance imaging; DCIS, ductal carcinoma in situ; IDC, invasive ductal carcinoma; NG, nuclear grade; ER, oestrogen receptor

^a^ANOVA, *t* test

^b^The size was confirmed by surgical specimens and the excluded tumours received neoadjuvant chemotherapy

## Discussion

dbPET has been developed to improve the detection of small breast cancers. One of the advantages of this study was the comparison of dbPET with other breast imaging modalities and the determination of breast cancer detectability, especially early breast cancer. In this study, the sensitivity of dbPET for all cases was 89%, similar to that of other reports (78–92%) [9, 11, 13, 19]. dbPET is a high-resolution molecular imaging machine for breast cancer with high sensitivity and specificity. In particular, the sensitivity of dbPET for early stage breast cancer was higher than that of WBPET/MRI. Kumar et al. [20] showed a sensitivity of 23% for primary breast cancers sized ≤ 10 mm using WBPET. The sensitivity of dbPET for DCIS was reported to be 41–90%, higher than that with WBPET [9, 13, 21]. In this study, the dbPET detection rate for DCIS was 81.8%, which was comparable to that of previous reports. US and MRI are associated with increased sensitivity for high-density breasts and reduced specificity [22]. The high-density breast was also related to the sensitivity of MMG. In this study, 15 lesions (total lesions scanned: 80) were undetected by MMG. In particular, 10 of the 15 lesions could be detected by dbPET. Thus, our findings indicated that dbPET could detect breast cancer that was undetected by MMG screening.

The FDG uptake of dbPET is significantly correlated with pathological and immunohistochemical factors [3–8]. In general, ILC shows a lower sensitivity and FDG uptake than IDC on FDG PET or WBPET/CT [3–5, 23–25]. In this study, two ILC cases were undetected by dbPET. In terms of immunohistology, poor prognostic factors, including a large tumour size (≥ 2 cm), negative ER status, negative PgR status, high Ki67%, and high histological grade, are correlated with high SUVmax [3–8]. In this study, the SUVmax of dbPET tended to be significantly higher, with negative ER status, higher grade, and higher Ki67% among the histopathological features. Furthermore, in most studies, triple-negative breast cancer (ER-/HER2-) showed the highest SUVmax compared with the other subtypes [3, 8, 23, 26–29]. The FDG uptake level is speculated to be one of the prognostic factors. As these results suggest accumulation of FDG as a sensitive biomarker of breast cancer tissue, it is expected to be applied to the evaluation of anticancer drug treatments [30, 31]. Based on these results, dbPET can assist in the detection of early stage breast cancers, systemic or contralateral breast screening, and prognosis prediction.

There is a certain limit to dbPET, depending on the detectable FOV and histopathological factors. The dbPET system takes an image with the patient lying prone with the breasts hanging down. Therefore, the areas near the chest wall tend to become blind areas. The limitation of FOV is attributed to the thickness of the breast and the location of the tumour on the patient’s side [21, 32]. In this study, five tumours were undetected by dbPET, despite detected by WBPET/MRI. It was presumed that the tumours were located outside the FOV. Of the five tumours, four tumours were located in the lateral area, and the BMI of the affected four patients was ≤ 20 kg/m^2^. We hypothesised that the cause was that the mat was not compressed due to less weight, and the breast could not hang down sufficiently. Therefore, we decided to improve the FOV based on the results.

We reduced the mat thickness of the detector in the middle of this study. First, we replaced the mat of the detector with a removable mat, which was used to relieve pressure and subsequent pain from the edge of the detector. After replacing the initial 20-mm mat with the 5-mm mat, we were able to secure a sufficient imaging range from the retro mammary space (Fig. 2). We also compared the emission scan time of dbPET, which ranged between 3 and 5 min, and confirmed that the detection ability was equivalent. In addition, the imaging range was expanded by adding a slight tilt to the imaging position. Finally, we could shorten the emission scan time and improve the patient position. The pain associated with scanning was also reduced, and the blind area could be further reduced by adjusting the patient position. We restarted the study using the thinner mat. After the improvement of the mat, scan time, and position, no tumour was located outside the FOV.

**Fig. 3:**
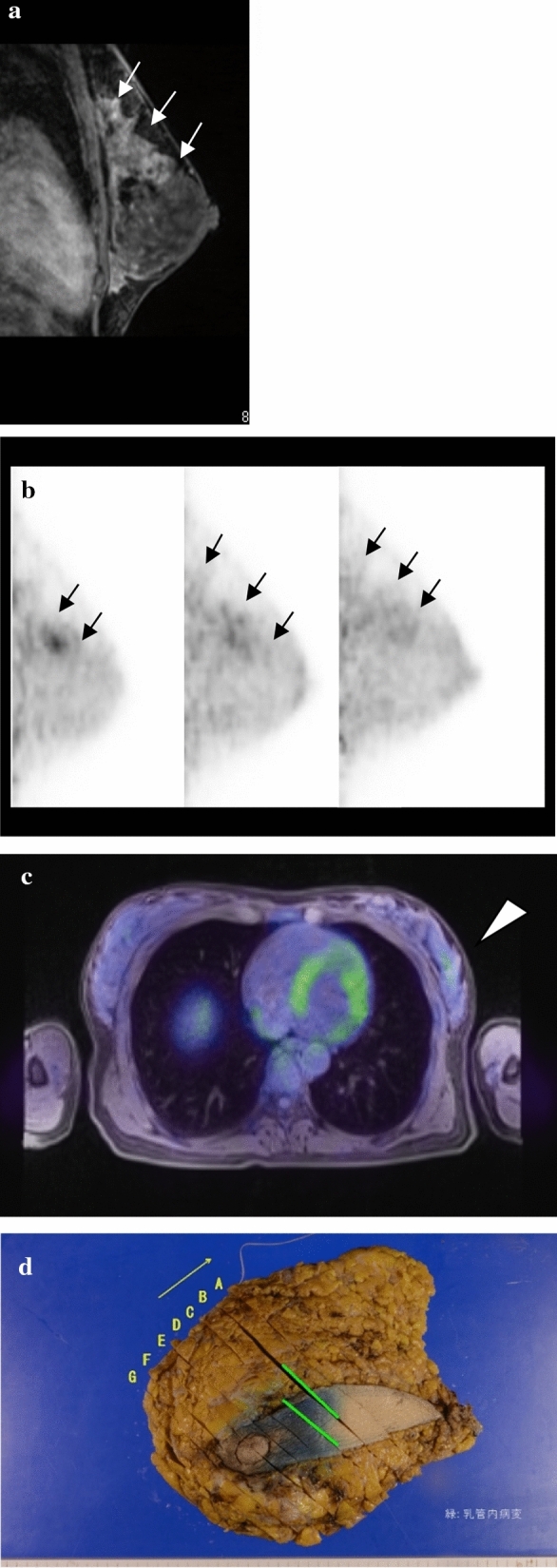
A representative case. **a** Sagittal MRI image. The contrast-enhanced area showed the spread of the DCIS. **b** Sagittal dbPET images. These images presented the segmental FDG uptake (SUVmax, 4.08). The FDG uptake areas were similar to the contrast-enhanced area of (**a**). **c** Axial WBPET/MR image. The FDG uptake was very weak and, thus, it was difficult to determine the spread of DCIS. **d** Surgical specimen and pathological result; DCIS with 5- × 2.5-cm size (green-shaded area); ER, 90%; PR, < 5%; HER2, 2+; NG2; Ki67, 20%. MRI, magnetic resonance imaging (MRI); DCIS, ductal carcinoma in situ; dbPET, dedicated breast positron emission tomography; FDG, F-18 fluorodeoxyglucose; SUVmax, maximum standard uptake value; WBPET/MRI, whole-body positron emission tomography/magnetic resonance imaging; ER, oestrogen receptor; PgR, progesterone receptor; HER2, human epidermal growth factor 2; NG, nuclear grade

dbPET can also be used to obtain tomographic images. We demonstrate a case, wherein it was possible to detect the spread of the intraductal breast cancer component, similar to that in MRI (Fig. 3). Thus, there is a possibility that dbPET can be used for screening as well as to evaluate the tumour extent for surgery.

However, the limitation of this study was that all lesions were diagnosed as breast cancers. We could not demonstrate the specificity of dbPET. Therefore, it is necessary to continue to investigate the specificity of dbPET.

In summary, dbPET could detect early breast cancers, some of which could not be detected on WBPET. Therefore, dbPET can be expected to become one of the preferred breast cancer screening modalities. Moreover, the FDG uptake level on dbPET can reflect the biology of breast cancer.
